# Brain health in the clinical practice of Spanish neurologists: a national cross‐sectional survey

**DOI:** 10.1002/alz.095726

**Published:** 2025-01-09

**Authors:** Carmen Lage‐Martinez, Raquel Gutierrez Zuniga, Sandra Giménez, Rafal Nowak, Jonathan Adrian Zegarra‐Valdivia, Neus Falgàs, Mircea Balasa, Brian A Lawlor, Iracema Leroi

**Affiliations:** ^1^ Marques de Valdecilla University Hospital, Santander, Cantabria Spain; ^2^ IDIVAL, Santander Spain; ^3^ Global Brain Health Institute, San Francisco, CA USA; ^4^ Trinity College Dublin, Dublin Ireland; ^5^ Global Brain Health Institute (GBHI), Trinity College Dublin, Dublin Ireland; ^6^ Alzheimer Down Unit, Barcelona Spain; ^7^ Multidisciplinary Sleep unit. Hospital de la Santa Creu i Sant Pau, Institut d'Investigació Biomèdica Sant Pau (IIB SANT PAU), Barcelona Spain; ^8^ Atlantic Fellow for Equity in Brain Health. Global Brain Health Institute, San Francisco, CA USA; ^9^ Center of Biomedical Investigation Network for Neurodegenerative Diseases (CIBERNED), Madrid Spain; ^10^ Sant Pau Memory Unit, Hospital de la Santa Creu i Sant Pau, Biomedical Research Institute Sant Pau, Universitat Autònoma de Barcelona, Barcelona Spain; ^11^ Achucarro Basque Center for Neuroscience, Leioa Spain; ^12^ Global Brain Health Institute (GBHI), University of California San Francisco, San Francisco, CA USA; ^13^ CIBERNED, Madrid Spain; ^14^ Unitat d'Alzheimer i Altres Trastorns Cognitius, Servei de Neurologia, Hospital Clínic, Barcelona Spain; ^15^ Global Brain Health Institute, Trinity College, Dublin Ireland; ^16^ Hospital Clinic, Barcelona Spain; ^17^ Global Brain Health Institute, Trinity College Dublin, Dublin Ireland

## Abstract

**Background:**

Brain Health (BH) perspectives and practices among health care professionals constitute a crucial piece of information for developing tailored initiatives to foster BH at the regional level. We aim to characterize knowledge and clinical practices associated with BH in Spanish neurologists by a cross‐sectional survey.

**Method:**

An original online survey was distributed by email among all members of the Spanish Society of Neurology. The survey included four sections: BH knowledge; BH‐related clinical practices; perspectives about the importance of BH in neurology practice; and limiting factors for BH promotion in daily practice.

**Result:**

400 neurologists participated in the survey, and 75.8% fully completed it. Participants were 61.7% females, mainly workers in tertiary (71.0%) and public hospitals (85.7%), and 74.2% had a sub‐specialization. Many participants considered to be familiar (41.7%) or somehow familiar (43.6%) with the concept and recommendations of BH, but 48.7% did not know any supporting scientific study. A substantial percentage of respondents considered that BH cannot be improved in aging (19.2%) or dementia (21.1%). Clinical interview was the main tool to evaluate BH (81.3%), but 22.1% of neurologists did not routinely explore BH. A higher percentage of females than males declared to regularly ask patients about their familiar situation (68.9% vs 53.0%, p 0.018) and social activities (62.9% vs 47.8%, p 0.019) and recommended more frequently healthy diet (75.7% vs 64.9%, p 0.0080). Also, more females considered that it is important to develop BH plans (98.9% vs 89.4% males), even adjusting by age (p <0.0001). The main limiting factor for BH evaluation and promotion during clinical visits were time constraints (in 76.8% of cases), followed by scarcity of support resources (61.3%), but a significant number of neurologists with <10 years of experience considered lack of education about official BH recommendations as a limitation (26.5% vs 14.0% in >20 years, p 0.047).

**Conclusion:**

This survey was conducted as a situational analysis to guide next steps of the BH roadmap in Spain. While time constraints and scarcity of resources should be approached from a health policy perspective, we identify poor BH knowledge as an opportunity to improve BH related practices, especially among young neurologists.

